# Mitochondrial Quality Control and Cell Death

**DOI:** 10.3390/ijms262211084

**Published:** 2025-11-16

**Authors:** Zurui Zhang, Mengyuan Zhang, Hongchi Jin, Shuang Lv, Yilei Li, Yanru Li

**Affiliations:** 1 The Key Laboratory of Pathobiology, Ministry of Education, College of Basic Medical Sciences, Jilin University, 126 Xinmin Avenue, Changchun 130021, China; zrzhang9923@mails.jlu.edu.cn (Z.Z.); mengyuanz23@mails.jlu.edu.cn (M.Z.); jinhc24@mails.jlu.edu.cn (H.J.); lvshuang@jlu.edu.cn (S.L.); liyl@jlu.edu.cn (Y.L.); 2 The First Norman Bethune College of Clinical Medicine, Jilin University, Changchun 130021, China

**Keywords:** mitochondrial quality control, mitochondrial dynamics, mitochondrial fission, mitochondrial fusion, mitochondrial autophagy, pyroptosis, ferroptosis, apoptosis

## Abstract

Mitochondrial quality control includes mitochondrial biogenesis, fusion, fission (to maintain mitochondrial function), and mitochondrial autophagy (for removing damaged mitochondria). This is a highly delicate and complex process involving many molecules. Mitochondrial quality control is crucial for maintaining mitochondrial homeostasis and function, preserving energy supply, eliminating damaged mitochondria to prevent cytotoxicity, promoting mitochondrial regeneration and repair, protecting cells from oxidative stress and senescence, and facilitating cellular communication and material exchange. In this review, we introduce the structure and function of mitochondria, the mechanisms of quality control, and the relationship between mitochondrial quality control and cellular processes such as pyroptosis, apoptosis, and ferroptosis. We also summarize the proteins, enzymes, and their molecular mechanisms involved in these processes and propose a “spatiotemporal-threshold” model for the mitochondrial quality control–cell death axis.

## 1. Introduction

Mitochondria are essential organelles in eukaryotic cells, often referred to as the energy factory of the cell. The function is to produce adenosine triphosphate (ATP) through oxidative phosphorylation, thereby providing energy for cellular activities. Mitochondria were first observed under a microscope by the German biologist Richard Altmann in 1890, who initially referred to them as “biological body”. In 1897, Benda officially designated them as “mitochondria” originated from a symbiotic event between archaea and eukaryotes, during which free-living bacteria were engulfed by ancestral eukaryotic cells and gradually evolved into organelles.

Mitochondria possess a double-membrane structure: the outer membrane is thin and smooth, directly interfacing with the cytoplasm, while the inner membrane is extensively folded to form cristae. This folding increases the surface area of the membrane, thereby facilitating energy conversion processes. The lumen enclosed by the inner membrane is referred to as the matrix, which contains mitochondrial DNA, ribosomes, and various enzymes. The primary functions of mitochondria are energy metabolism, supplying cells with energy through oxidative phosphorylation [1]; the tricarboxylic acid cycle occurring in the matrix, which breaks down sugars, fatty acids, and amino acids to release energy [2]; the release of reactive oxygen species and apoptosis-associated proteins to regulate cell death [2]; participating in the removal of metabolic wastes such as pyruvate and ammonia [3]; and contributing to intracellular calcium ion storage and release, thereby influencing cellular signalling [4].

Mitochondrial quality control is a crucial mechanism for maintaining mitochondrial function and cellular homeostasis. This process encompasses several key components, including mitochondrial biogenesis, mitochondrial dynamics, mitochondrial autophagy, and mitochondrial DNA repair. These mechanisms collectively ensure normal functioning in energy metabolism, calcium ion homeostasis, redox reactions, and signaling by regulating mitochondrial health [5]. Mitochondria play a significant role in cell death through various pathways, including apoptosis, pyroptosis, and ferroptosis. They can release apoptosis-inducing factors, such as cytochrome c, and activate caspase family proteins to initiate programmed cell death. Additionally, mitochondria can release inflammatory factors, such as IL-1β, which contribute to the inflammatory response leading to pyroptosis. Furthermore, they can trigger ferroptosis through iron-dependent lipid peroxidation during the mitochondrial tricarboxylic acid cycle and oxidative phosphorylation [6].

## 2. Structure of Mitochondria

**Figure 1:**
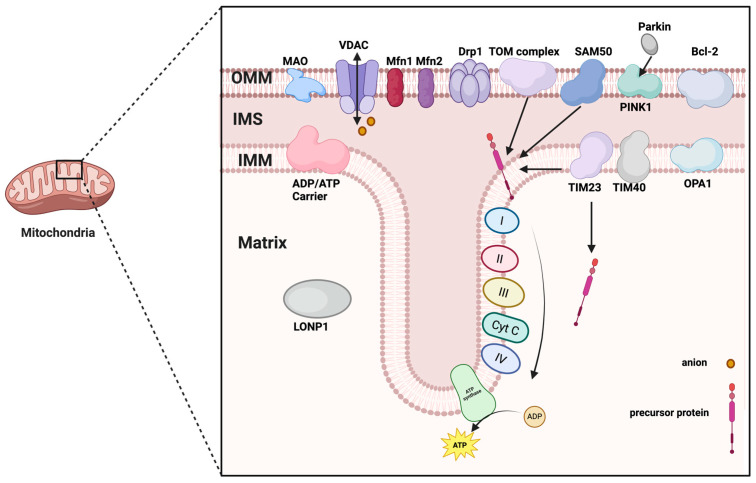
The structure of mitochondria. Abbreviations: OMM, outer mitochondrial membrane; IMS, mitochondrial intermembrane space; IMM, inner mitochondrial membrane; MAO, monoamine oxidase; VDAC, voltage-dependent anion channel; Mfn1/2, mitofusin 1/2; Drp1, dynamin-related protein 1; SAM50, the key subunit of SAM complex; PINK1, PTEN-induced putative kinase 1; Bcl-2, B-cell lymphoma-2 family of proteins; TIM23/40, Translocase of inner mitochondrial membrane 23/40; OPA1, Optic atrophy protein 1; LONP1, lon peptidase 1; Cyt c, Cytochrome c. (Created in BioRender. zhang, m. (2025) https://BioRender.com/6tdbqfl).

Mitochondria are closed vesicular organelles encased in a double membrane, typically circular or ovoid in shape. They consist of an outer membrane, an inner membrane, an outer lumen, and a matrix (Figure 1). The outer membrane is smooth and highly permeable, allowing water, electrolytes, and small molecules to pass through. In contrast, the inner membrane is highly impermeable and folds inward to form numerous structures known as cristae. The space between the outer and inner membranes is referred to as the outer lumen, which is also highly permeable and facilitates the passage of ions and small molecules. The area between the inner membrane and the cristae is a densely concentrated environment of proteins and lipids known as the stroma, which contains mitochondrial DNA (mtDNA), ribosomes, and a variety of enzymes.

### 2.1. Outer Membrane

The outer mitochondrial membrane serves as an effective selective permeability barrier and an exchange platform for substance transport [7]. It permits the passage of substances with molecular weights less than 5000 Da, enabling small molecules and specific metabolites to move freely in and out of the mitochondria. This selective permeability protects the cell from harmful mitochondrial products, including reactive oxygen species and immunogenic mtDNA [8].

The mitochondrial outer membrane contains a diverse array of proteins that perform a wide range of functions, including material transport, signaling, apoptosis regulation, and the maintenance of mitochondrial structure. These proteins collaborate to sustain cellular energy metabolism and physiological functions through interactions with other organelles, such as the endoplasmic reticulum and nuclear membrane. Notable proteins found in the mitochondrial outer membrane include the Tom complex, Bcl-2 associated X protein (Bax), B-cell lymphoma-2 family of proteins (Bcl-2), mitofusin 1/2 (Mfn1/2), monoamine oxidase, nod-like receptor protein 3(NLRP3), and Bcl-2/adenovirus E1B 19kDa interacting protein 3 (BNIP3), among others. Pore-forming proteins, such as the Tom complex, facilitate the entry of specific proteins into the mitochondria [9]. The Bcl-2 family of proteins is expressed on the mitochondrial outer membrane, where Bax proteins can influence the release of cytochrome c by modulating the permeability of the outer mitochondrial membrane in response to cellular damage or apoptotic signaling [10,11]. Additionally, monoamine oxidase and other proteins participate in critical biochemical reactions at the outer membrane [12]. Mfn1/2 proteins, localized in the outer mitochondrial membrane, play a role in regulating mitochondrial fusion [13]. The voltage-dependent anion channel (VDAC) is primarily responsible for regulating the transmembrane transport of ions and small molecules, thereby influencing intracellular signaling and metabolic activities, particularly in calcium regulation [14]. NLRP3 is an inflammation-associated protein located on the mitochondrial outer membrane, involved in immune responses and the activation of inflammatory vesicles [15]. BNIP3 is implicated in the regulation of mitochondrial autophagy and apoptosis, while FUN14 domain-containing 1 (FUNDC1) is associated with iron metabolism, playing a role in the transport and storage of iron ions. NADH dehydrogenase (ubiquinone) 1 alpha subcomplex subunit 9(NDUFA9) is closely related to the function of the mitochondrial electron transport chain [10]. Dynamin-related protein 1(Drp1) is a core driver of mitochondrial fission. It binds to receptor proteins such as mitochondrial fission 1 protein (Fis1), fission factor (Mff) and dynamics proteins (MiD49, MiD51) to form a multimeric ring structure, which ultimately leads to the rupture of the mitochondrial membrane [16]. When mitochondria are damaged, the PTEN-induced putative kinase 1(PINK1) protein is activated and stabilized on the outer membrane. It undergoes autophosphorylation, which promotes the recruitment of Parkin and the ubiquitination process [17].

**Figure 2:**
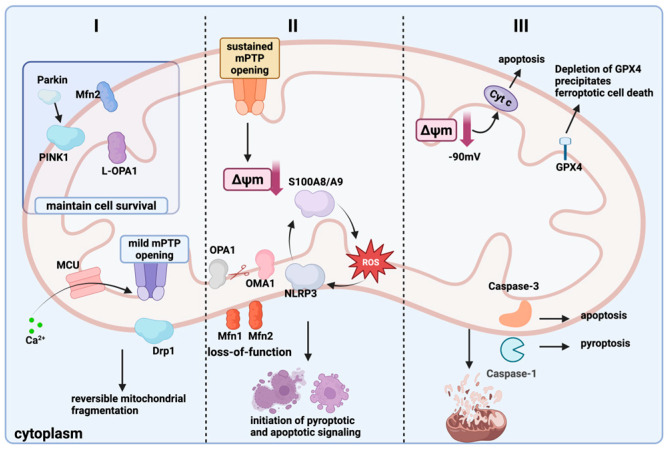
The mitochondrial quality control–cell death axis “spatiotemporal-threshold” model. Grade I (Partial Reversible Stage): The voltage-dependent anion channel (VDAC) on the mitochondrial outer membrane and the mitochondrial calcium uniporter (MCU) on the inner membrane take in some Ca^2+^ into the mitochondria. A brief calcium pulse causes the mitochondrial permeability transition pore (MPTP) to open slightly, recruiting Drp1 to the outer membrane and inducing reversible fragmentation of the mitochondrial network [18,19,20]. Grade II (Threshold Critical Stage): Sustained calcium overload drives prolonged MPTP opening, causing a steep decline in mitochondrial membrane potential (Δψm). OMA1 is activated and cleaves OPA1, resulting in Mfn1/2 inactivation and failed fusion. Meanwhile, the positive feedback loop of S100A8/A9-ROS-NLRP3 is amplified, triggering pyroptosis and apoptosis signals in parallel [21,22,23]. Grade III (Irreversible Stage): When Δψm declines to the “point-of-no-return”, cytochrome c is massively released into the cytoplasm; if GPX4 activity is depleted, this leads to ferroptosis, whereas if Caspase-1/3 predominates, pyroptosis or apoptosis ensues. Mitochondrial autophagy fails due to p62 depletion, leading to the accumulation of mitochondrial fragments. This ultimately drives the cell death program [24]. (Created in BioRender. zhang, m. (2025) https://BioRender.com/jt8v64j).

Thus, the outer membrane VDAC-Mfn2-Drp1 not only maintains mitochondrial shape but also acts as a “sentinel sensor” for mitochondrial quality control. Any imbalance in its conformation directly determines the threshold level of the first calcium ion barrier (Figure 2).

### 2.2. Inner Membrane

The inner mitochondrial membrane is abundant in proteins, widely distributed, and plays a crucial role in various physiological processes, including energy metabolism, ATP synthesis, electron transport, and mitochondrial division and fusion. It is central to mitochondrial energy metabolism. Additionally, the inner mitochondrial membrane is rich in cardiolipin (CL) and deficient in cholesterol, a combination that contributes to the establishment of the proton gradient and the maintenance of the electrochemical potential difference.

The inner mitochondrial membrane consists of three specialized regions: the inner boundary membrane (IBM), cristae junctions (CJs), and cristae [25]. Compared to the outer membrane, the inner boundary membrane is thicker and has a higher protein content. It folds inward to form cristae, which increase the membrane’s surface area and enhance the energy conversion efficiency of mitochondria [26,27]. The inner boundary membrane contains a significant number of enzymes involved in oxidative phosphorylation [28]. The inner boundary membrane is interconnected with the matrix by cristae, forming CJs These junctions are believed to prevent diffusion between the inner space of the cristae and the outer lumen by sealing off the contents of the cristae and regulating the controlled transfer of solutes, metabolites, and proteins. The electron transport chain complexes located on the cristae junctions include NADH dehydrogenase, succinate dehydrogenase, cytochrome c reductase, cytochrome c oxidase, and proton pumps, all of which are integral to the electron transport process in mitochondria [29,30,31]. Additionally, the inner mitochondrial membrane contains a significant number of potassium channels, calcium channels, and sodium–potassium pumps, which are responsible for ion transport and the regulation of concentration gradients [29]. The SecYEG complex facilitates the transport of polypeptide chains from the inner membrane to the outer membrane [32]. The Tom complex (comprising Tom20 and Tom40) is responsible for transporting proteins synthesized by ribosomes to the inner mitochondrial membrane [33]. The TIM complex (consisting of Tim23 and Tim40) is involved in the transmembrane transport of proteins [33]. Chaperonin-containing TCP1 complex (CCT) and the key subunit of SAM complex (SAM50) proteins play roles in the folding and modification of proteins on the inner mitochondrial membrane [34]. Optic atrophy protein 1(OPA1) is an optic atrophy protein that is primarily located in the inner mitochondrial membrane. It promotes the fusion of the inner membrane through changes in its own conformation [35].

The inner membrane cardiolipin-OPA1-L-OPA1 axis serves as a “double safeguard” for energy and fusion. Once it is cleaved by OMA1 into S-OPA1, this safeguard is removed, marking the critical point of the second-level threshold structural switch (Figure 2).

### 2.3. Outer Lumen

The mitochondrial outer lumen, located between the outer and inner membranes, is a complex region that serves as a crucial conduit for material exchange. The proteins within this space primarily include pore proteins, transporter proteins, enzymes, and VDACs, all of which collectively contribute to functions such as material transport, energy metabolism, signaling, and the regulation of apoptotic cell death. Signal-anchored proteins bind to the outer lumen through specific structural domains and play a role in protein localization and transport [36]. Integrins encompass pore proteins, β-barrel proteins, and α-helical transmembrane fragment proteins, which are involved in mitochondrial protein transport, the regulation of mitochondrial dynamics, and apoptosis [37]. Through pore and transporter proteins, the outer lumen regulates the transport of molecules such as ATP, NADH, and CoA. Proteins like VDAC are critical in apoptosis and calcium signaling; during apoptosis, the permeability of VDAC increases, leading to the release of apoptosis-inducing factors, such as cytochrome c, into the cytoplasm. Additionally, the outer lumen contains fatty acid prolongase, adrenergic oxidase, and tryptophan degradation enzymes, which are involved in biochemical processes such as lipid metabolism, redox reactions, and amino acid degradation [38,39,40], thereby supporting mitochondrial energy production. Proteins located in the outer lumen may play a role in the quality control of mitochondrial proteins by removing defective proteins through ubiquitination and proteasomal degradation mechanisms [41].

The Drp1 oligomerization platform in the outer space and the Mff receptor provide the “physical scissors.” The assembly speed of this complex determines whether mitochondrial fragments can be cleared in time after a calcium pulse. This process is the rate-limiting step for the transition from the reversible grade I to the critical grade II (Figure 2).

### 2.4. Mitochondrial Matrix

Most mitochondrial proteins in the mitochondrial matrix are encoded by nuclear genes and enter the mitochondria through a single post-translational modification and translocation mechanism. The mitochondria’s DNA encodes only a small number of proteins, which are primarily involved in mitochondrial structure and function.

The mitochondrial matrix contains a diverse array of enzymes that play crucial roles in various metabolic processes, including key enzymes involved in the tricarboxylic acid cycle, fatty acid oxidation, and amino acid metabolism. These enzymes catalyze biochemical reactions within the mitochondria and are essential for energy metabolism. ATP/ADP transport proteins, known as AAC, help regulate energy homeostasis by facilitating the exchange of ATP from the cytoplasm for ADP in the mitochondrial matrix. Additionally, the mitochondrial matrix houses subunits of the oxidative phosphorylation complex, which are integral to the function of the electron transport chain [42]. DNA repair proteins, such as LONP1 (a homohexamer involved in protein degradation and DNA quality control), are also present in the mitochondrial matrix [43]. Proteins within the mitochondrial matrix are typically imported via a precursor protein import system; for instance, the TIM23 complex is responsible for translocating precursor proteins from the intermembrane space to the matrix [44].

The matrix LONP1 and the TIM23 precursor import system together create a “protein quality checkpoint.” When LONP1 becomes overwhelmed by excessive oxidized substrates, the matrix protein homeostasis collapses, becoming the structural root cause for autophagy depletion and the irreversible grade III (Figure 2).

The four-level structure (outer membrane, inner membrane, intermembrane space, and matrix) is not a static “compartment,” but a dynamic physical carrier for quality control (Table 1). The outer membrane VDAC/Mfn2 determines the “entry gate,” the inner membrane OPA1/CL forms the “energy seal,” the intermembrane space Drp1 acts as the “scissors”. The matrix LONP1 is responsible for “waste recycling.” When these structural modules become misaligned in time or space or imbalanced expression, the cell enters the three progressive stages described below, eventually leading to pyroptosis, ferroptosis, or apoptosis.

## 3. Mitochondrial Quality Control

Mitochondrial mass regulation is a crucial mechanism for maintaining mitochondrial health and cellular homeostasis. Its primary objective is to ensure the relative stability of mitochondrial number, morphology, and function through various pathways. This regulation encompasses mitochondrial biogenesis, dynamics, autophagy, protein hydrolysis, and the formation of mitochondria-derived vesicles (Table 2). By maintaining a dynamic balance between the quantity and quality of mitochondria, mitochondrial mass regulation plays a vital role in cellular homeostasis and health. It supports cellular energy metabolism, prevents oxidative stress, and regulates cell survival and apoptosis. Additionally, it significantly influences the onset and progression of various diseases. A comprehensive study of the effects of mitochondrial mass regulation on cell death can enhance our understanding of disease pathogenesis and aid in the development of new therapeutic strategies.

### 3.1. Mitochondrial Biogenesis

Mitochondrial biogenesis is a complex and dynamic process that involves multiple activities, including the co-expression of mitochondrial and nuclear DNA, protein synthesis, and the fusion and division of mitochondria. This process meets the energy requirements of cells under various physiological conditions by increasing both the quantity and quality of mitochondria. Mitochondrial biogenesis is essential for cell growth, differentiation, and survival. From an evolutionary perspective, the formation of mitochondria is the result of endosymbiotic events that provide host cells with an efficient means of energy production, thereby driving the complexity of life forms.

Nuclear respiratory factors 1 (NRF-1) and 2 (NRF-2) are essential transcription factors involved in mitochondrial biogenesis, regulating mitochondrial gene expression by binding to the promoter regions of mitochondrial genes [45]. PPAR-gamma coactivator 1alpha (PGC-1α) is another crucial regulator that enhances the expression of mitochondrial genes by activating NRF-1 and NRF-2 [46]. AMP-activated protein kinase (AMPK), a key sensor of the cell’s energy status, is activated during energy deficits, which subsequently promotes mitochondrial biogenesis by upregulating the expression of PGC-1α [47]. Mitochondrial biogenesis is also influenced by various physiological and pathological conditions; for instance, exercise, cold exposure, and oxidative stress significantly impact mitochondrial quantity and function [48]. In certain disease states, such as acute inflammation or sepsis, mitochondrial biogenesis may be stimulated to restore impaired mitochondrial function [49]. This process increases both the number and functionality of mitochondria, thereby enhancing the cell’s energy metabolism to meet the energy demands of physiological activities.

### 3.2. Mitochondrial Division

Mitochondrial division is a crucial dynamic process within the cell that maintains mitochondrial function and quality control, thereby preventing the detrimental effects of damaged mitochondria on overall cellular performance. By promoting mitochondrial autophagy, smaller mitochondria produced through division can be degraded via the autophagy pathway, effectively removing damaged mitochondrial components. This process also regulates cellular energy metabolism, allowing cells to adapt to energy demands by modulating the number and distribution of mitochondria. Mitochondrial division in high-pressure environments can promote apoptosis, release reactive oxygen species, and participate in cellular stress responses and signaling. During the cell cycle, mitochondrial division facilitates the even distribution of mitochondria to daughter cells, ensuring that new cells have an adequate energy supply. By regulating the number and distribution of mitochondria, mitochondrial division maintains cellular energy levels, thereby meeting energy requirements in various physiological states [50]. When cells are subjected to metabolic stress or injury, mitochondrial division can assist in the removal of damaged mitochondria, preventing further cellular damage [51]. Additionally, mitochondrial division is closely associated with apoptosis; fragmented mitochondria formed during this process can trigger apoptotic signals and promote cell death. This mechanism plays a crucial role in cell development, tissue remodeling, and immune responses [52]. In neural differentiation and synapse formation, mitochondrial division is essential for providing sufficient ATP to support these processes [53].

There are two types of mitochondrial fission: intermediate fission and peripheral division. Intermediate fission occurs in the central region of the mitochondria and is typically associated with healthy mitochondrial proliferation. This type of fission does not exhibit abnormal physiological properties, such as decreased membrane potential or elevated levels of reactive oxygen species during the process. Intermediate fission is primarily mediated by Drp1, and actin, which interacts with the endoplasmic reticulum, also plays a role in this process. In contrast, peripheral division occurs near the ends of the mitochondria and is generally associated with damaged or dysfunctional mitochondria. During peripheral division, the mitochondrial membrane potential decreases, and levels of reactive oxygen species are significantly elevated. This division results in the formation of small mitochondria that often lack mtDNA, and these smaller mitochondria are subsequently degraded by lysosomes [54].

The molecular mechanism of mitochondrial division is complex and involves the interaction of multiple proteins. Drp1 is a key driver of mitochondrial division, forming a multimeric ring structure by binding to receptor proteins such as Fis1, Mff, MiD49, and MiD51, which ultimately leads to the rupture of mitochondrial membranes [16]. Fis1, a receptor protein located on the outer membrane of mitochondria, interacts with Drp1 and is capable of recruiting Drp1 independently, thereby promoting its recruitment and multimerization [55]. Mff aggregates directly at mitochondrial constrictions, with its intrinsic properties determining the extent of this aggregation [56]. MiD49 and MiD51 facilitate the oligomerization and activation of Drp1 through their interaction with it. Once activated, Drp1 self-assembles into ring-like structures with the assistance of receptor proteins such as Fis1 [57]. Drp1 then localizes to the mitochondrial fission site with the assistance of the endoplasmic reticulum and the actin cytoskeleton, initiating the contraction of the ring structure. Drp1 is activated by GTP, which induces significant elongation and rotation of its GTPase structural domains, as well as bundling of signaling elements and connection of hinge loops. This process forms a multivalent network of interactions that facilitates the polymerization of mitochondrial kinetic proteins MiD49 and MiD51 with mitochondrial monofilaments. Following GTP dissociation, the GTPase structural domain and bundled signaling elements of Drp1 dissociate, leading to the shortening of mitochondrial monofilaments and the coiling of the Drp1 dimer into a narrow, closed ring structure, ultimately completing mitochondrial division [57,58]. Additionally, AMPK can phosphorylate the Ser155 and Ser173 sites of Drp1, while Extracellular signal-regulated kinase (ERK) phosphorylation of the S616 site of Drp1 promotes mitochondrial division and tumor growth. The ERK inhibitor FAK reduces phosphorylation at the S616 site of Drp1 in cardiomyocyte recipients [59,60]. Furthermore, the mitochondria-endoplasmic reticulum contact sites play a crucial role in mitochondrial division and influence this process by regulating endoplasmic reticulum pressure [61].

### 3.3. Mitochondrial Fusion

Mitochondrial fusion plays a crucial role in maintaining the morphological and functional balance of mitochondria by merging the outer and inner membranes of two mitochondria into a single, larger organelle. This process enhances the membrane potential, reduces oxidative stress, promotes glucose metabolism and autophagy, and protects cells from damage. Additionally, mitochondrial fusion facilitates the exchange of substances between mitochondria, including lipids, proteins, and mtDNA, which is essential for maintaining the functional and genetic stability of mitochondria and meeting cellular energy demands [62]. Furthermore, mitochondrial fusion aids in the repair of damaged mitochondrial DNA, which is vital for cell survival during ischemia–reperfusion injury. In the development of specialized cells, such as oocytes, mitochondrial fusion increases ATP levels to support cell maturation and division [63].

Mfn1 and Mfn2 are essential mitochondrial fusion factors that belong to the GTPase family. They facilitate the remodeling of the mitochondrial outer membrane and promote the fusion of the inner membrane through GTP hydrolysis. These two proteins possess multiple conserved structural domains, including a GTPase-binding region, a high-affinity HR1 region, a transmembrane region, and a low-affinity HR2 region. The HR1 and HR2 regions can form dimers, which are crucial for outer membrane fusion [64]. Mfn1 primarily mediates outer membrane fusion, while Mfn2 is involved in inner membrane fusion. The N-terminal end of Mfn2 contains a RAS-like structural domain, which enhances its activity compared to Mfn1 in certain contexts [65]. OPA1, an optic atrophy protein, is primarily localized in the inner membrane of mitochondria and facilitates the fusion of the inner membrane through its conformational changes. There are several isomeric forms of OPA1, including long (L) and short (S) isoforms, each playing distinct roles in mitochondrial fission and fusion activities, with L-OPA1 predominantly involved in mitochondrial fusion and S-OPA1 in mitochondrial fission [35]. In mammals, the localization of OPA1 to just one of two separate mitochondria is sufficient to drive the fusion of their membranes [66]. The process of mitochondrial fusion begins with Mfn1 mediating the contact and fusion of the outer membranes of two neighboring mitochondria, a process that requires GTP hydrolysis to facilitate membrane polymerization and fusion [67,68]. Subsequently, OPA1 acts on the inner membrane to promote fusion through changes in its conformation. The short peptide form of OPA1 promotes mitochondrial fusion, whereas its long peptide form inhibits this process, ultimately resulting in the formation of a larger mitochondrial structure [69]. Mitochondrial fusion enhances the integrity of the mitochondrial inner membrane by merging two mitochondria into a larger entity, thereby supporting the energy metabolic needs of the cell [70].

### 3.4. Mitochondrial Autophagy

Mitochondrial autophagy is a critical regulatory mechanism that maintains cellular homeostasis and balances energy metabolism by selectively removing damaged or dysfunctional mitochondria. When mitochondria become impaired—due to factors such as the accumulation of reactive oxygen species, nutrient deficiency, or cellular senescence—their membrane potential becomes depolarized, which is a prerequisite for initiating mitochondrial autophagy [71]. Damaged mitochondria are encapsulated by a double membrane, forming a double-membrane autophagosome. This process is mediated by proteins such as Sequestosome 1(p62/SQSTM1), NIP3-like protein X (NIX), among others. The double-membrane autophagosomes then fuse with lysosomes to form mature autophagic lysosomes, where the mitochondria are degraded. The resulting mitochondrial components are broken down by lysosomal acid hydrolases, releasing recyclable molecules that contribute to the material cycle [72].

There are three pathways of mitochondrial autophagy: the ubiquitin-dependent pathway, the ubiquitin-independent pathway, and the other factor-regulated pathway. The ubiquitin-dependent pathway consists of two main signaling modes. The first is the PINK1/Parkin signaling axis. PINK1 is a protein expressed on the outer membrane of mitochondria. Under normal physiological conditions, mitochondrial proteases continuously degrade PINK1 to prevent its accumulation on the membrane. However, when mitochondria become depolarized, PINK1 accumulates on damaged mitochondria, activates its E3 ubiquitin ligase activity through autophosphorylation, and recruits Parkin to the damaged mitochondria [73,74]. Parkin, an E3 ubiquitin ligase, attaches ubiquitin tags to mitochondrial proteins such as Mfn2, VDAC1, and Miro when recruited by PINK1 [75]. These ubiquitinated proteins are subsequently recognized by autophagy receptors such as Optineurin (OPTN), nuclear dot protein 52 (NDP52), and microtubule-associated protein 1 light chain 3 (LC3). The ubiquitination of proteins on damaged mitochondria promotes their degradation [76]. The second pathway is mediated by receptor molecules such as NIX and prohibitin 2 (PHB2), which facilitate the entry of damaged mitochondria into the autophagosome by binding to ubiquitinated substrates on these mitochondria. PHB2 activates mitochondrial autophagy through the recruitment of the PARL-PAGAM5-PINK1 complex along with Parkin, and it promotes mitochondrial clearance by directly interacting with LC3 via the LIR structural domain [77]. Additionally, PHB2 enhances autophagy by inhibiting PARL activity, thereby protecting PINK1 from degradation and maintaining the mitochondrial membrane potential [78]. NIX is a receptor protein located in the inner mitochondrial membrane that becomes ubiquitylated under conditions of cellular stress. It subsequently accumulates at the outer mitochondrial membrane, inducing mitochondrial depolarization [79]. NIX is activated through the recruitment of PINK1, which binds directly to LC3-II via its LIR structural domain, thereby recruiting autophagy-associated proteins and promoting the encapsulation and degradation of damaged mitochondria [80]. When mitochondria are damaged, PHB2 is exposed on the outer membrane and binds directly to LC3-II, further promoting mitochondrial autophagy [81].

The first of the ubiquitin-independent pathways is mediated by proteins such as NIX, BNIP3, and PHB2, which bind directly to damaged mitochondria and promote their entry into the autophagosome through various mechanisms. For instance, BNIP3 facilitates the clearance of damaged mitochondria by interacting with BCL-2 family proteins and inhibiting their anti-apoptotic functions [82]. The second pathway is mediated by ULK1 kinase, which is activated during the initiation phase of autophagy. ULK1 kinase aids in the recognition and encapsulation of damaged mitochondria by binding to the ATG14 complex [78]. Among other factor-regulated pathways, LC3 serves as a key marker during autophagy, recognizing and binding to damaged mitochondria to facilitate their entry into the autophagosome. When cells experience an energy crisis, AMPK is activated, which in turn activates the kinase activity of ULK1 by phosphorylating the Thr172 site of ULK1, enabling it to further phosphorylate downstream substrates [83]. ULK1 activation can phosphorylate mitochondria-associated substrates, such as Atg13 and FIP200, thereby promoting the formation of the autophagy complex [84]. Additionally, ULK1 can enhance autophagy by phosphorylating the mitochondrial outer membrane protein FUNDC1, promoting its binding to LC3 and facilitating the recognition of damaged mitochondria. ULK1 also phosphorylates Parkin at Ser65, thereby enhancing Parkin’s function [85]. Reactive oxygen species (ROS) and c-Jun N-terminal kinase (JNK) can also trigger mitochondrial autophagy. Mitochondrial autophagy plays a crucial role in maintaining energy metabolism and regulating apoptosis. In neurons, mitochondrial autophagy is essential for removing damaged mitochondria and preserving neuronal function. In tumor cells, the regulation of mitochondrial autophagy can inhibit tumor cell proliferation.

**Table 2:** Molecular mechanisms of mitochondrial quality control.

Quality Control Processes	Proteins/Molecules	Molecular Mechanism	Reference
mitochondrial biogenesis	NRF1, NRF2	Key transcription factor, regulates mitochondrial gene expression	[46]
	PGC-1α	Activation of NRF1 and NRF2 for transcription of mitochondrial genes	[47]
	AMPK	Sensing cellular energy status, upregulating PGC-1α expression upon activation, and promoting mitochondrial biogenesis	[48]
mitochondrial division	Drp1	Core driver that binds to receptor proteins such as Fis1, Mff, MiD49, and MiD51, causing mitochondrial membrane rupture	[16]
	Fis1	Mitochondrial outer membrane receptor protein that recruits Drp1 and promotes its multimerization	[55]
	Mff	Aggregates at mitochondrial contractions and promotes mitochondrial division	[56]
	MiD49, MiD51	Interacts with Drp1 to promote its oligomerization and GTPase activity and facilitates division	[57]
	AMPK	Phosphorylation of Ser155 and Ser173 sites of Drp1 promotes mitochondrial division	[60]
	Erk	Phosphorylation of the S616 site of Drp1 promotes mitochondrial division	[61]
mitochondrial fusion	Mfn1	Core factor of outer membrane fusion, mediates outer membrane contact and fusion	[65]
	Mfn2	Core factor of endothelial fusion, involved in endothelial fusion, more active than Mfn1	[65]
	OPA1	A key protein in endosomal fusion that promotes endosomal fusion through conformational changes	[66]
	S-OPA1	Promotes OPA1-CL binding and membrane fusion	[72]
mitochondrial autophagy	PINK1	Accumulates on damaged mitochondria, activates E3 ubiquitin ligase activity, and recruits Parkin	[80]
	Parkin	E3 ubiquitin ligase, ubiquitinates mitochondrial proteins and promotes their degradation	[82]
	p62/SQSTM1	Mediates entry of damaged mitochondria into autophagosomes	[78]
	NIX	Ubiquitination aggregates at the outer mitochondrial membrane, induces depolarization and promotes mitochondrial autophagy	[86]
	BNIP3	Interacts with BCL-2 family proteins to inhibit their anti-apoptotic function and promote damaged mitochondrial clearance	[87]
	ULK1	Activation phosphorylates downstream substrates to promote autophagy complex formation and mitochondrial autophagy	[88]
	AMPK	Sensing energy states, activating ULK1, and promoting mitochondrial autophagy	[88]
	ROS, JNK	Trigger mitochondrial autophagy	[88]

## 4. Regulation of Mitochondrial Mass and Cell Death

There is a close relationship between the regulation of mitochondrial mass and cell death, which is evident in the central role of mitochondria in cellular energy metabolism, structural stability, and cell death signaling (Table 3). Regulating mitochondrial mass is a crucial mechanism for maintaining mitochondrial function and cellular homeostasis. This includes processes such as mitochondrial dynamics, autophagy, and mitochondrial DNA repair, which collectively regulate mitochondrial number, morphology, and function to ensure proper operation under various physiological and pathological conditions. Apoptosis is typically associated with an increase in the permeability of the outer mitochondrial membrane. Ferroptosis, a form of regulated cell death, is closely linked to mitochondrial dysfunction and is characterized by a loss of mitochondrial membrane potential and the production of reactive oxygen species. Additionally, the release of inflammatory factors from mitochondria activates inflammatory vesicles, which can trigger focal cell death.

In addition, mitochondrial permeability transition (MPT) is a key event in the mitochondrial stress response, characterized by the opening of non-selective pores in the inner mitochondrial membrane, known as the mitochondrial permeability transition pore (MPTP). This leads to calcium ion efflux, collapse of the membrane potential, and release of apoptotic factors. MPT is closely related to mitochondrial dynamics and autophagy, which are important mechanisms for quality control. It serves as a crucial link between calcium imbalance and cell death [7].

Current research focuses on individual modes of cell death yet lacks a systematic exploration of how these three types of programmed cell death are interconnected within mitochondria. Accordingly, this review proposes the mitochondrial quality control–cell death axis “spatiotemporal-threshold” model (Figure 2), which categorizes mitochondrial quality control failure into three progressive stages. Using calcium signaling as a common timer and the duration of MPTP opening as a fate switch, this model elucidates the logic behind the allocation of different death modes.

### 4.1. Mitochondrial Quality Control and Cell Pyroptosis

Cellular pyroptosis is a form of programmed cell death first proposed by Cookson and Brennan in 2001. It describes a pro-inflammatory type of cell death mediated by inflammatory vesicles. This process is characterized by cell swelling, membrane rupture, and the release of inflammatory factors, distinguishing it significantly from other modes of cell death, such as apoptosis and necrosis [86]. The central mechanism of cellular pyroptosis involves the cleavage and activation of Gasdermin D (GSDMD) proteins. In the classical pathway, Caspase-1 is activated to cleave GSDMD, resulting in the formation of pore-forming GSDMD-N. This leads to increased cell membrane permeability and the release of cellular contents, triggering an inflammatory response [89]. Conversely, the non-classical pathway relies on the cleavage of Caspase-4/5 or Caspase-3, which also induces cell lysis by activating GSDMD [90].

Mitochondrial quality regulation plays a central role in cellular pyroptosis, with mitochondrial dysfunction promoting this process through mechanisms such as the release of ROS, increased membrane permeability, and the activation of inflammatory vesicles. Concurrently, the regulation of mitochondrial division, fusion, autophagy, and other processes can effectively inhibit the onset of cellular death. Mitochondrial dysfunction leads to the accumulation of excessive ROS, which can activate inflammatory vesicles such as NLRP3, thereby promoting the release of inflammatory mediators like IL-1β and IL-18, further triggering focal cell death [87,88]. Increased permeability of the mitochondrial outer membrane facilitates the release of apoptotic factors, such as cytochrome c, from the mitochondria into the cytoplasm, activating caspase-3 and other caspases to initiate a cascade of reactions that ultimately lead to cellular pyroptosis [91]. Additionally, mitochondrial calcium overload contributes to mitochondrial dysfunction, exacerbating ROS production and activating inflammatory vesicles [92]. In addition, the emerging regulators S100A8/A9 also play a significant role in mitochondrial dysfunction, particularly in cardiovascular pathology [93] and sepsis models [94]. Inflammation-associated proteins S100A8/A9 may act as key upstream triggers of pyroptosis by inducing mitochondrial ROS production and MPTP opening, thereby activating the NLRP3 inflammasome [23]. Mitochondrial damage triggers autophagy, which helps maintain the stability of the intracellular environment by removing damaged mitochondria; however, excessive autophagy may instead exacerbate cellular pyroptosis [95]. Mitochondrial fission aids in the separation and segregation of damaged mitochondria, while mitochondrial fusion facilitates the repair of these damaged organelles [96]. Mitochondrial autophagy serves as a crucial quality control mechanism, and the inhibition of this process can worsen cell death.

Mitochondrial calcium overload can induce the opening of the MPTP, which further exacerbates mitochondrial membrane potential loss and ROS release, forming a positive feedback loop. This process activates the NLRP3 inflammasome and promotes the execution of pyroptosis mediated by GSDMD [97]. Research indicates that inhibiting MPTP release (such as through the use of cyclosporine A) significantly reduces the degree of pyroptosis, suggesting that MPTP plays a central role in pyroptosis.

During cellular pyrolysis, mitochondrial morphology gradually transitions from the typical oval shape to a solid, irregular, and severely damaged state. This process is accompanied by the loss of mitochondrial cristae, a decrease in membrane potential, and increased membrane permeability [98]. In certain cells, such as differentiated myofibroblasts, the mitochondria are encapsulated within muscle fibers and appear relatively quiescent and granular. In contrast, in other cell types, the mitochondria form a predominantly linear network structure.

In summary, pyroptosis can be regarded as the “inflammatory exit” for the transition from grade II to grade III: calcium overload and sustained MPTP opening provide the initial signal, while the S100A8/A9-ROS axis determines the intensity of NLRP3 activation. Drp1-mediated fragmentation and autophagy exhaustion jointly lock GSDMD cleavage, thereby transforming mitochondrial crisis into membrane perforation and inflammation.

### 4.2. Mitochondrial Quality Control and Ferroptosis

Ferroptosis is a form of cell death that relies on iron ions and lipid peroxidation. It is characterized by alterations in mitochondrial morphology, the accumulation of ROS, and the buildup of lipid peroxides. This phenomenon was first proposed in 2012 by Brent R. Stockwell’s laboratory at Columbia University and was formally defined as a type of non-apoptotic cell death in the same year [99]. Morphologically, mitochondria may decrease in size or even disappear, mitochondrial cristae may diminish, mitochondrial membrane density may increase, and the outer membrane may rupture [100]. Cytoplasmic components condense into swellings, while the nucleus remains normal in size, with no chromatin aggregates present. The cell membrane ruptures, leading to the release of cellular contents [101].

Mitochondria generate ROS through the electron transport chain. These ROS not only damage the mitochondria themselves but also affect the redox state of other cellular components [102]. For instance, ferroptosis inducers such as Erastin and RSL3 can inhibit mitochondrial ROS production, thereby preventing ferroptosis. Additionally, antioxidant enzymes in mitochondria, including GPX4, SOD2, and MGST1, can scavenge ROS and protect cells from oxidative damage [103]. Intramitochondrial iron content accounts for 20–50% of total cellular iron. While iron is an essential cofactor for mitochondrial function, excess iron can trigger abnormal ROS production or enzymatic reactions that lead to lipid peroxidation. For example, Erastin promotes ferroptosis by enhancing mitochondrial membrane potential and increasing ROS production [104]. MtDNA damage is a significant trigger of ferroptosis. When mtDNA is compromised, it activates innate immune responses, such as the STING/TLR9 pathway, which subsequently induces ferroptosis [104]. The regulation of mitochondrial mass is crucial for maintaining the stability of mitochondrial function and structure; cellular susceptibility to ferroptosis increases when mitochondria are damaged. In type I ferroptosis, mitochondrial dysfunction and ROS production play a pivotal role, whereas in type II ferroptosis, the protective function of the GPX4 gene is essential, with mitochondrial dysfunction serving a secondary role [24]. Mitochondria involved in ferroptosis typically exhibit a reduction in size, an increase in mitochondrial membrane density, a decrease or complete loss of cristae, rupture of the outer membrane, and a significant reduction in the overall number of mitochondria. Notably, the number and length of cristae change significantly, with 55.7% of mitochondria categorized by a cristae length of 2.5–3 µm or a cristae count of 6–12, compared to a lower percentage observed in control samples [105].

Ferroptosis represents the “oxidative exit” at the grade III threshold: when GPX4 cannot counteract the surge of MPTP-ROS, S100A8/A9 further chelates free iron, forming an “iron-ROS positive feedback loop.” Mitochondrial fragments accumulate due to autophagy failure, and lipid peroxidation spreads, ultimately crossing the irreversible oxidative threshold.

### 4.3. Regulation of Mitochondrial Mass and Apoptosis

Apoptosis is a form of programmed cell death characterized by distinct morphological and biological features. During apoptosis, the nuclei of mid-stage cells undergo densification, with chromatin condensing either at the nuclear membrane or in the center of the nucleus in the early stages. This process causes the cell to adopt shapes resembling crescents, petals, or rings. In the later stages, the nucleus may fragment into smaller nuclear components [106]. Early in the process, mitochondria increase in size and exhibit enhanced cristae, indicating mitochondrial proliferation; subsequently, these proliferating mitochondria may undergo vacuolization. A hallmark of apoptosis is the formation of apoptotic bodies, which are membrane-bound vesicles containing cytoplasmic, organelle, and nuclear debris [107]. Additionally, DNA is cleaved into oligonucleotide fragments of approximately 180–200 bp [108]. Importantly, apoptosis typically does not elicit an inflammatory response, thereby helping to maintain tissue homeostasis.

Mitochondria typically maintain a dynamic balance of morphology and function through processes of fusion and fission; however, this balance is disrupted during apoptosis. In the apoptotic process, mitochondrial fission is enhanced while fusion is diminished. The Bcl-2 family of proteins plays a crucial role in this regulation, with certain members promoting mitochondrial fission and others, such as Bcl-XL, inhibiting it [109]. A decrease in mitochondrial membrane potential is a significant indicator of apoptosis. As the mitochondrial membrane potential declines, ATP synthesis is reduced, ROS levels increase, and the permeability of the outer mitochondrial membrane rises. This change facilitates the release of apoptotic signaling factors, such as cytochrome c, into the cytoplasm. These apoptotic factors further activate caspases, including Caspase-9 and Caspase-3, thereby triggering the apoptotic cascade [110]. It has been demonstrated that in the early stages of apoptosis, the structure of the mitochondrial network deteriorates, and the mitochondrial cristae undergo remodeling. This process leads to a transformation in mitochondrial morphology from a tubular to a granular form, which may involve phenomena such as splitting or fusion, along with cristae remodeling [98].

Mitochondrial fragmentation is a hallmark of apoptosis, occurring approximately one hour after the induction of apoptosis and continuing to increase over time. This fragmentation manifests as a transition from elongated shapes to shorter or more rounded forms. Drp1 is a key regulator of mitochondrial fragmentation; it promotes this process by interacting with proteins such as Fis1 and OPA1. Calcium ion overload induces sustained MPTP opening, leading to irreversible loss of mitochondrial membrane potential and massive release of cytochrome C into the cytoplasm, thereby activating the Caspase-9/Caspase-3 cascade. Concurrently, MPT-induced mitochondrial fragmentation (Drp1 recruitment) further amplifies the apoptotic signal, forming the ‘calcium-MPT-apoptosis’ axis. The overexpression of Drp1 or its dominant-negative mutants (e.g., K38A) significantly enhances mitochondrial fragmentation while inhibiting the release of cytochrome c and the execution of apoptosis [111].

In cardiovascular diseases, cardiomyocyte apoptosis is closely associated with hypertension, ischemia–reperfusion injury, and other cardiovascular conditions. Studies have demonstrated that Angiotensin II (Ang II) induces mitochondrial fragmentation in cardiomyocytes through the activation of Drp1, thereby exacerbating apoptosis In neurodegenerative diseases such as Alzheimer’s disease and Parkinson’s disease, mitochondrial dysfunction and abnormal morphology lead to neuronal apoptosis, which is a significant contributing factor [112]. Additionally, abnormal regulation of mitochondrial dynamics in cancer cells may enhance their proliferation and anti-apoptotic capacity. The knockdown or inhibition of Drp1 significantly reduces the proliferation of cancer cells and increases their apoptosis.

Apoptosis is the most classic “protease-type exit” at the third level: After the MPTP remains continuously open and the Δψm collapses, excessive release of cytochrome c occurs; if the Caspase-9/3 cascade is irreversibly activated, the mitochondrial network fragments into granules, initiating the cell death program.

**Table 3:** Mitochondrial quality regulation and cell death.

Type of Cell Death	Quality Control Processes	Mechanism of Action	Proteins and Pathways	Reference
cellular pyroptosis	Increased outer membrane permeability Increased mitochondrial fission and decreased fusion Inhibition of mitochondrial autophagy	Promote cellular pyroptosis Isolates and repairs damaged mitochondria Loss of mitochondrial cristae and increased membrane permeability	GSDMD, Caspase-1, NLRP3, IL-1β, Mitochondrial calcium overload	[88,89,90]
iron death	Mitochondria decrease in size, cristae disappear Rupture of the outer mitochondrial membrane Inhibition of mitochondrial autophagy and activation of innate immune response	Lipid peroxidation, ROS accumulation Loss of membrane potential Induced occurrence of iron death	Erastin, RSL3, GPX4, SOD2, MGST1, mitochondrial membrane potential, STING/TLR9	[99,100,103,104]
apoptosis	Enhanced mitochondrial division, reduced fusion, fragmentation Mitochondria become larger, cristae increase, then undergo vacuolization and change from tubular to granular MPT-induced calcium imbalance	Decreased ATP synthesis and increased ROS content Cytochrome C release Calcium overload; opening of the MPTP; loss of membrane potential; release of cytochrome c	Bcl-2, Bcl-XL, Caspase9, Caspase3, Drp1, Fis1, OPA1	[106,107,109,110]

## 5. Summary

Mitochondria, often referred to as the “energy factories”, generate adenosine triphosphate (ATP) through oxidative phosphorylation, providing essential energy for cellular functions. In addition to energy production, mitochondria are involved in various biological processes, including cell signaling, calcium ion homeostasis, and programmed cell death. Consequently, the quality and functionality of mitochondria are crucial for the survival and activity of cells. Mitochondrial quality regulation encompasses processes such as mitochondrial biogenesis, division and fusion, and mitochondrial autophagy. This regulation is a highly intricate and delicate process that relies on the coordinated action of numerous molecules. Maintaining mitochondrial homeostasis and function is vital for ensuring a consistent energy supply, eliminating damaged mitochondria to prevent cytotoxicity, promoting mitochondrial regeneration and repair, protecting cells from oxidative stress and aging, and facilitating cellular communication and material exchange. Mitochondria also play a significant role in apoptosis, pyroptosis, and ferroptosis. Therefore, the regulation of mitochondrial mass can influence cell fate, providing valuable insights into the mechanisms underlying cell death.

This review provides introductions to the mitochondrial structure and function, as well as the mechanisms of quality control. Based on this, an integrated model is proposed, and the relationships between the model and different ways of cell death are reviewed and summarized. These may provide some insights and support for further research into the specific mechanisms underlying the relationship between mitochondrial quality control and cell death. Although the central role of mitochondria in cell death has been widely recognized, current research still faces multiple contradictions that require further research and experimental validation. For instance, while some studies indicate that a mitochondrial membrane potential (Δψm) reduction of ≤30% can activate MPTP and release cytochrome c, in sympathetic neurons or HeLa cells, prolonged survival or completion of the apoptotic program persists even with Δψm declines exceeding 50% or complete dissipation. This suggests that Δψm reduction alone does not constitute a universal switch determining cell death. More critically, existing experimental systems have systematic biases and technical blind spots. Traditional microscopy imaging cannot capture the sub-second dynamics that determine mitochondrial fate. Meanwhile, Δψm probes, such as TMRE, are prone to quenching and phototoxicity interference in high-density cells or tissue sections, leading to an overestimation of the “membrane potential drop threshold.”

Currently, there is a lack of clarity regarding the specific mechanisms underlying mitochondrial fragmentation, and it remains uncertain whether the processes and molecular mechanisms involved in mitochondrial fragmentation are consistent with those of mitochondrial division. Although mitochondria exhibit distinct molecular mechanisms in ferroptosis, pyroptosis, and apoptosis, there are no characteristic morphological changes associated with these processes. The extent to which the molecular mechanisms governing mitochondrial morphological changes can directly influence intracellular calcium ion levels and peroxide levels requires further investigation. Improving cell fate by modulating mitochondrial quality and function remains an important research direction.

In the future, research on mitochondrial quality control will progress from “describing correlations” towards “mechanism-intervention-prediction”. We may boldly propose a hypothesis: The rate at which OPA1 is cleaved by OMA1 may serve as a grade II threshold timer across cell types and can be kinetically regulated by small-molecule allosteric modulators, thereby determining whether cells cross the irreversible death threshold. This hypothesis may be realized through optogenetic-proton pump or gradient FCCP techniques, small-molecule allosteric modulator screening, and model validation. Finally, in translational medicine, multiple preclinical studies have provided clear therapeutic doses and safety windows, such as PLGA-Mdivi1 nanoneedles reducing myocardial infarction area in mice, AAV2-PINK1 rescuing dopaminergic neurons in primate PD models, and Ligustrazine nano-drug delivery systems targeting the PIEZO1-PHB2 axis to protect cardiomyocyte mitochondria. These mark the gradual transition of mitochondrial quality control research towards clinical trials.

## Figures and Tables

**Table 1:** Mitochondrial structure and protein function.

Structure	Protein	Function	Reference
OMM	TOM complex	Transfer of specific proteins	[9]
	BAX	Increased mitochondrial outer membrane permeability activates apoptotic program	[10]
	Monoamine oxidase	Catalytic oxidation and deamination of monoamines	[12]
	Mfn1/2	Involved in the regulation of mitochondrial fusion	[13]
	VDAC	Transporting ions and small molecules	[14]
	Bcl-2	Regulation of apoptosis	[11]
	NLRP3	Involved in immune response and activation of inflammatory vesicles	[15]
	BNIP3	Involved in the regulation of mitochondrial autophagy and apoptosis	[11]
	FUNDC1	Involved in the transportation and storage of iron ions	[11]
	NDUFA9	Closely related to the function of the mitochondrial electron transport chain	[11]
	Drp1	Causes the rupture of the mitochondrial membrane	[16]
	PINK1	Promotes Parkin recruitment and ubiquitination	[17]
IMM	ATPase	Drive ATP synthesis	[32]
	NADH dehydrogenase	Oxidizes NADH to NAD+ and generates electron flow	[32]
	Succinate dehydrogenase	Oxidizing succinic acid to fenugreek acid and transferring electrons	[32]
	Cytochrome C reductase	Accepts electrons from complex II and passes them to cytochrome C	[32]
	Cytochrome C oxidase	Accepts electrons delivered by cytochrome c and delivers them to oxygen to produce water	[32]
	Proton pump	Driving ATP synthesis through a proton gradient	[32]
	SecYEG complex	Transport polypeptide chains from the inner membrane to the outer membrane	[32]
	TOM complex	Transport of proteins synthesized by ribosomes in the inner mitochondrial membrane	[33]
	TIM complex	Involved in transmembrane transport of proteins	[33]
	CCT, SAM50	Involved in protein folding, modification and quality control	[34]
	OPA1	Promotes the fusion of the inner membrane	[35]
Outer Lumen	signal-anchored	Involved in protein localization and transport	[36]
	porin	Perform small molecule transport	[37]
	β-barrel protein	Bound to TIM for translocation to the matrix	[37]
	alpha helical transmembrane fragment protein	Embedded in the outer membrane, involved in protein transport, quality control and membrane dynamics	[37]
	fatty acid elongase	Lengthening of short-chain fatty acids into longer saturated fatty acids maintains cellular lipid homeostasis and energy metabolism	[38]
	adrenaline oxidase	Involved in the oxidative deamination of adrenaline	[39]
	tryptophan degrading enzyme	Catalyzes the breakdown of serine to 5-HT or kynurenine	[40]
Mitochondrial Matrix	AAC	Regulation of energy homeostasis by exchange of ATP in the cytoplasm and ADP in the mitochondrial matrix	[42]
	LONP1	Involved in protein degradation and DNA quality control	[43]
	TIM23	Transport of precursor proteins from the mitochondrial membrane space to the matrix	[44]

## Data Availability

No new data were created or analyzed in this study. Data sharing is not applicable to this article.
